# A novel procedure on next generation sequencing data analysis using text mining algorithm

**DOI:** 10.1186/s12859-016-1075-9

**Published:** 2016-05-13

**Authors:** Weizhong Zhao, James J. Chen, Roger Perkins, Yuping Wang, Zhichao Liu, Huixiao Hong, Weida Tong, Wen Zou

**Affiliations:** Division of Bioinformatics and Biostatistics, National Center for Toxicological Research, U.S. Food and Drug Administration, 3900 NCTR Road, HFT-20, Jefferson, AR 72079 USA; College of Information Engineering, Xiangtan University, Xiangtan, Hunan Province, China

**Keywords:** Data mining, Topic modeling, Next-generation sequencing (NGS), Genetic diversity, Biomarker

## Abstract

**Background:**

Next-generation sequencing (NGS) technologies have provided researchers with vast possibilities in various biological and biomedical research areas. Efficient data mining strategies are in high demand for large scale comparative and evolutional studies to be performed on the large amounts of data derived from NGS projects. Topic modeling is an active research field in machine learning and has been mainly used as an analytical tool to structure large textual corpora for data mining.

**Methods:**

We report a novel procedure to analyse NGS data using topic modeling. It consists of four major procedures: NGS data retrieval, preprocessing, topic modeling, and data mining using Latent Dirichlet Allocation (LDA) topic outputs. The NGS data set of the *Salmonella enterica* strains were used as a case study to show the workflow of this procedure. The perplexity measurement of the topic numbers and the convergence efficiencies of Gibbs sampling were calculated and discussed for achieving the best result from the proposed procedure.

**Results:**

The output topics by LDA algorithms could be treated as features of *Salmonella* strains to accurately describe the genetic diversity of *fliC* gene in various serotypes. The results of a two-way hierarchical clustering and data matrix analysis on LDA-derived matrices successfully classified *Salmonella* serotypes based on the NGS data. The implementation of topic modeling in NGS data analysis procedure provides a new way to elucidate genetic information from NGS data, and identify the gene-phenotype relationships and biomarkers, especially in the era of biological and medical big data.

**Conclusion:**

The implementation of topic modeling in NGS data analysis provides a new way to elucidate genetic information from NGS data, and identify the gene-phenotype relationships and biomarkers, especially in the era of biological and medical big data.

**Electronic supplementary material:**

The online version of this article (doi:10.1186/s12859-016-1075-9) contains supplementary material, which is available to authorized users.

## Background

Next generation sequencing (NGS) [1] is a term that refers to post-Sanger sequencing methods. The primary advantage offered by NGS technologies over traditional sequencing methods is the production of large volumes of sequence data inexpensively and with a high degree of flexibility for the level of resolution required for given experiments. The production of large numbers of low-cost, high-quality sequences has enabled the scientific community to address an increasingly diverse range of biological and medical problems, including clinical diagnostics [2, 3], epidemiological investigation [4], species classification and gene discovery in metagenomics studies [5], virology [6] and genomic analysis [7].

Although NGS technologies are increasingly used in many areas, the large amounts of data produced by NGS technologies present a significant challenge for data analysis and interpretation. Advanced high-performance computing and intensive bioinformatics support is essential for the successful application of NGS technologies. Currently, a variety of analysis tools and software have been developed for the early stage of NGS technologies. Most of these tools are used for the general categories, such as sequence alignment, genome assembly and annotation, and genetic variation detection [8]. Limited research has been reported on data mining strategies for large next generation sequencing data to address biology-driven questions.

Topic modeling is an active research technique in machine learning that has wide analytical applicability for interpreting large data sets in text mining [9–11] and image retrieval procedures [12, 13]. The basic idea in topic modeling is that a document is a mixture of latent topics, each of which is expressed by a distribution on words. Latent Dirichlet Allocation (LDA) [10] is the most popular topic modeling algorithm. In an enhanced version of the earlier models [14, 15], LDA uses two Dirichlet-Multinomial distributions to model both the relationships between documents and topics and between topics and words. Two probability matrices are provided by the LDA approach: 1. per-document topic distributions and 2. per-topic word distributions. Approximate methods, such as variational inference [10] and Markov chain Monte Carlo (MCMC) [16], are commonly used in LDA analysis to calculate the posterior probabilities. The calculated probability matrices are used to make inference about the topics and documents for text mining. Topic modeling has been applied for various purposes, such as protein structure representation [17], FDA drug labeling [18] and metagenome data analysis [19], however, we are not aware of any research that has been reported which applies the text mining algorithms to next generation sequence data analysis.

In this study, we propose a procedure that applies LDA topic modeling to analyze NGS data, especially those on the large sequence datasets. The NGS data set containing the *Salmonella fliC* gene was used as a case study to show the workflow and the function of this procedure. The *fliC* gene encodes a *Salmonella* phase 1 antigen, and is considered one of the *Salmonella* serotype determinant genes [20]. The developed procedure was applied to the *fliC* gene-containing NGS sequences of 119 *Salmonella* strains of nine *Salmonella* serotypes. These sequences were retrieved from the database of the National Center for Biotechnology Information (NCBI) and/or other sequence databases, and were transformed into the files of documents on which the LDA algorithm was run and two matrices were generated. The two matrices were then analyzed by hierarchical clustering and other data mining methods to elucidate the hidden information within the content of DNA sequences.

Based on our limited knowledge, the proposed method in this study is the first attempt of applying topic modeling to NGS data analysis at the level of phenotype-determinant genes. Better performance and accuracy was observed when comparing this method with Hamming distance method [21] by clustering analysis and classification. The results showed that the topic modeling provides a promising novel approach to analysis of NGS data for the purpose of understanding and decoding hidden genetic information in a biological system.

## Methods

### Dataset construction

In this study, the whole genome sequences of 119 strains (Additional file 1: Table S1) of *Salmonella* O antigen group B [22] were retrieved from the NCBI database, including 75 strains of *S*. Agona, 14 strains of *S*. Heidelberg, one strain of *S*. Paratyphi B, two strains of *S*. Saintpaul, two strains of *S*. Schwarzengrund, one strain of *S*. Stanley, 22 strains of *S*. Typhimurium, one strain of *S*. Typhimurium var.5-, and one strain of *S*. 4, 12:i:- [23]. The dataset was constructed and preprocessed by our developed pipeline [24] as briefly described in the following: The retrieved sequence reads or contigs were collected to a data pool. For each strain, the sequence fragment best matching the reference *fliC* gene was selected by blasting with the serotype-specific reference *fliC,* using the Basic Local Alignment Search Tool (BLAST) [25]. In this study, the reference *fliC* genes used for the strains of *S*. Agona, *S*. Heidelberg, *S*. Paratyphi B, *S*. Saintpaul, *S*. Schwarzengrund and *S*. Stanley were those of *S*. Agona SL483, *S*. Heidelberg SL476, *S*. Paratyphi B SPB7, *S*. Newport SL254, *S*. Schwarzengrund CVM19633 and *S*. Typhi CT18, respectively. The strains of *S*. Typhimurium, *S*. Typhimurium var.5- and 1 *S*. 4, 12:i:- [26] used the *fliC* of *S.* Typhimurium LT2 as the reference gene. All the reference genes were retrieved from NCBI by the same pipeline. The metadata of the *fliC* gene-containing NGS data of 119 *Salmonella* strains are listed in Additional file 1: Table S1.

### NGS data preprocessing

**Fig. 1:**
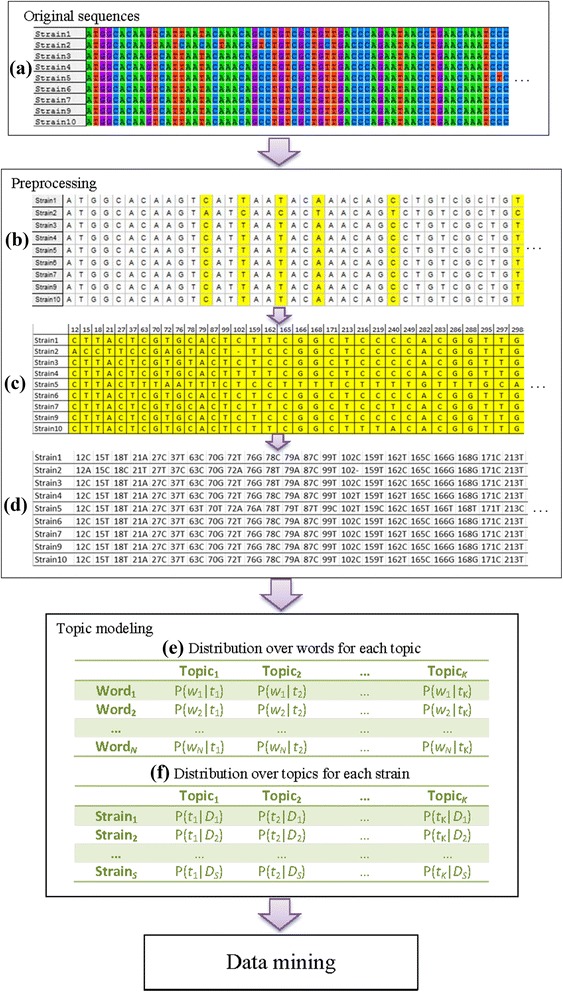
Flowchart of the proposed procedure

The procedures for data preprocessing are shown in Fig. 1(b-d). The sequences in the constructed dataset (Fig. 1(a)) were aligned by an algorithm of multiple sequence alignment (MSA), such as MUSCLE [27] or CLUSTAL [28], generating a dataset of aligned sequences (including insertions and deletions, or indels) (Fig. 1(b)). The nucleotide differences at each site, called single nucleotide polymorphisms (SNPs), were collected and are shown in Fig. 1(c). Each of the 119 strains had its corresponding file of words consisting of both SNPs and the locations of the SNPs in the sequences (Fig. 1(d)).

### Topic modeling

After preprocessing, a text corpus was generated in which each of the documents corresponded to one of the 119 strains, and all the documents had the same number of words. The LDA program implemented in Mallet [29] was utilized to model the corpus to get the latent topics and the topic mixture distribution for each strain.

**Fig. 2:**
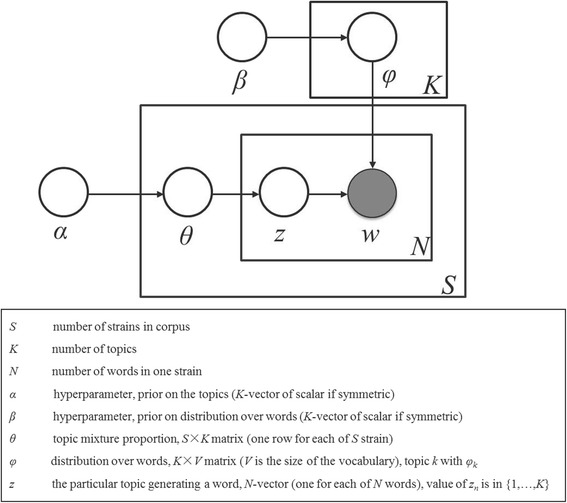
Graphical representation of the LDA model. The shaded circle represents the observed variable and white circles represent latent variables

LDA is a generative probabilistic model whose graphical model representation is shown in Fig. 2. LDA generates a given corpus according to the following process [10]:

1. For each topic *k,* where *k* in {1… *K*}, pick a distribution over words *φ*_*k*_ ~ *Dir*(*β*);
2. For each strain *D*_*s*_, where *s* in {1… *S*},
  - Pick a distribution over topics *θ*_*s*_ ~ *Dir*(*θ*);
  - For each word *w*_*n*_ with *n* in {1… ***N***},
    1. Pick a topic *z* ~ *Multinomial* (*θ*_*s*_);
    2. Pick word *w*_*n*_ ~ *Multinomial* (*φ*_*z*_);

In the generative process, *Dir* represented a Dirichlet distribution and *Multinomial* represented a Multinomial distribution. The distributions of words for topics and distributions of topics for documents were viewed as random variables obeying Dirichlet distributions with parameters *β* and *α,* respectively. Words in documents were treated as random variables obeying the Multinomial distribution of topics.

Given a corpus of *S* strains *D* = {*D*_1_, *D*_2_,…, *D*_*S*_}, the probability of the corpus:

1 $$ p\left(D\left|\alpha, \beta \right.\right)={\displaystyle \prod_{s=1}^s\int p\left({\theta}_s\left|\alpha \right.\right)}\left({\displaystyle \prod_{n=1}^N{\displaystyle \sum_{z=1}^kp\left({z}_{sn}\left|{\theta}_s\right.\right)p\left({w}_{sn}\left|\varphi {z}_{sn}\right.\right)p\left(\varphi z\left|\beta \right.\right)}}\right){d}_{\theta s}{d}_{\theta z} $$

In this study, Gibbs sampling [11], a special case of Markov chain Monte Carlo (MCMC) [16] approach, was used to sample posterior distribution of *θ*_*s* '_*Z*_*sn*_ and *φz*_*sn*_.The LDA algorithm was run on the corpus several times with different number of topics. For all the runs, we assigned *α* = 0.1 and *β* = 0.01 as initial values for the two hyper-parameters, and 2000 iterations in Gibbs sampling. The default values were applied for the other parameters in the LDA model in Mallet.

### Perplexity measurement

Leave-one-out cross-validation was used to measure the perplexity of LDA algorithm. The LDA algorithm was first trained by 118 samples, and then the obtained LDA model was applied to calculate the perplexity of the left-out sample. This process was repeated 119 times until each of the samples had been left out once. The average perplexity of the 119 samples was taken as the final perplexity for the corresponding number of topics.

### Data mining

#### Strain characterization

The per-document topic distributions and the per-topic word distributions were obtained after LDA processing. Words with high probability for given topics were selected to characterize and differentiate bacterial strains.

#### Two-way hierarchical clustering

Two-way clustering is a data mining technique which allows simultaneous clustering of the rows and columns of a matrix. In the two-way hierarchical clustering analysis in this study, the topic mixture distributions of strains were viewed as the strain representatives. The dissimilarities between strains and topics were calculated by Euclidean distance of topic mixture distributions of strains. The result describes simultaneously the subgroups of samples and the relationships among topics. Hierarchical cluster analysis using the complete linkage was applied on the dissimilarities to perform a two-way hierarchical clustering. Function “heatmap.2” in package “gplots” of R [30] was utilized to implement the two-way clustering. The colors from blue to red indicate the values of the topic probabilities of the strains ranging from 0 to 1.

#### Distance Matrix Analysis

In the distance matrix analysis, Euclidean distance measure was used to measure the dissimilarity between strains based on the strain-topic mixtures derived from the *fliC* SNPs dataset. The program “dist” in package “stats” of R (with default values of parameters) [31] was utilized to calculate the Euclidean distances between all the strains. The colors from blue to red represent the values of the Euclidean distances as they range from 0 to 1.

### Evaluation of topic modeling performance

To evaluate the performance of topic modeling, we applied clustering and classification algorithms on the sample-topic matrices (Fig. 1(f)) generated by LDA and similarity matrices generated by Hamming Distance [21], and the results were compared based on the serotypes (Additional file 1: Table S1) of the samples which were viewed as the sample’s true labels.

1. Hamming Distance measures
  The documents (samples) in Fig. 1(d) were transformed into matrix of Vector Space Model (VSM) [32] which is a commonly used model for representing text documents. In the VSM matrix, the number of row is the number of samples and the number of column represents the size of the vocabulary. Using Hamming Distance measures, the traditional clustering and classification methods were conducted and the results were compared with those conducted on the sample-topic matrices from LDA (Fig. 1(f)).

2. Cluster analysis and result comparison

3. Classification analysis and comparison
  Topic model-derived clustering method [33] was applied, in which LDA was utilized as a feature reduction approach for cluster analysis. The LDA-derived topics were considered as the new features of datasets. The sample-topic matrix (Fig. 1(f)) was treated as a new representation of the original dataset. Based on the sample-topic matrix (topic number was chosen as 5 and 30, respectively), conventional clustering algorithms, such as *k*-means, was used for the clustering analysis. The number of clusters was set as 7 in the *k*-means method due to 7 different serotypes in the dataset. While in comparison, *k*-means algorithm was also applied on VSM matrix using Hamming Distance similarities. For further comparison, due to the dimension reduction of topic modeling approach, the traditional tool of PCA was used to reduce features (Numbers of 2, 5, 10 and 30 were randomly selected as the reduced features, respectively) of VSM matrix followed by the *k*-means cluster analysis. Moreover, clustering by only LDA referred as “highest probable topic assignment” [33] (5 and 30 topics were used) was also used for comparison. In “highest probable topic assignment”, the LDA-derived topics were made as the clusters of the dataset. Then, each sample was assigned to the cluster (Topic) with the highest probability in the row of the sample-topic matrix. To interpret the clustering results obtained by the *k*-means algorithm, samples in each cluster were labeled as the dominant serotype of the samples in the cluster. The predicted labels of samples were compared with the true labels (serotypes) to evaluate the clustering quality.
  The clustering results were evaluated by Normalized mutual information (NMI) [34] and Adjusted Rand Index (ARI) [35]. NMI and ARI are two external validation metrics to evaluate the quality of clustering results with respect to the given true labels of datasets. The range of NMI and ARI values is 0–1. In general, the larger the value is, the better the clustering quality is.
  Two commonly used classification algorithms, Support Vector Machine (SVM) [36] and Random Forest (RF) [37], were applied on the sample-topic matrix obtained by LDA with the topic number set to 5 and 30, respectively. SVM and RF were also applied to the VSM matrix for comparison. Accuracy rate was used to evaluate and compare the classification results. Since classification is a supervised learning task, training dataset is required to include all of the true labels in the testing dataset. Therefore, the new data set for classification consists of 117 samples with two serotypes (true labels) Paratyphi B and Stanley samples removed from the original dataset due to the insufficient samples. Leave-One-Out Cross-Validation (LOOCV) was conducted on the 117 samples and the predicted accuracy rate was calculated. In this study, the function “svm” (with “Polynomial” kernel and default values of other parameters) in R package “e1071” and function “randomForest” (number of trees setting as 500 and default values of other parameters) in R package “randomForest” were utilized to train the classifiers.

## Results

In this study, we propose a novel procedure for applying the concept of topic modeling to the analysis and mining of NGS data. Assuming that the SNPs composition (four nucleotides and their locations) in NGS sequences can be considered and treated as “words”, the original sequence data are transformed to a file of “a bag of words”. A topic modeling procedure is then run on the document file to create two digital matrices, on which various data mining algorithms are applied to reveal the hidden genetic information in the sequences. A NGS data set of 119 *Salmonella* outbreak strains was used as a case study to show the workflow and its applications.

### Procedure

Figure 1 shows a schematic representation of the proposed procedure to transform the original sequence data to two digital matrices by topic modeling. The data set, first constructed (Fig. 1(a)) by our developed pipeline [24], is described in detail in “Methods”. The best *fliC* gene-matching sequences from all of the 119 *Salmonella* strains were collected in the dataset (Fig. 1(a)). Nucleotides A, T, G, and C are shown by different colors. In the procedures for data preprocessing (Fig. 1 (b-d)), the best *fliC* gene-matching sequences were multi-aligned and the variant nucleotides were found at 840 sites in 119 strains. All the nucleotides at the 840 sites in the 119 sequences are designated as SNPs in this study (displayed in yellow in Fig. 1(b)). The collection of all the SNPs in the dataset (Fig. 1(c)) was then transformed into a text corpus, in which each of the 119 *Salmonella* strains had its unique file of words (Fig. 1(d)). The final vocabulary size is 2379 and the obtained corpus had a total of 99,960 words (occurrences) from the 119 strains.

LDA algorithms run on the corpus and generated two digital matrices: Fig. 1(e) shows per-topic word distributions; and Fig. 1(f) exhibits per-document topic distributions. The two matrices provided vast information pools available for data mining.

### Topic analysis

**Fig. 3:**
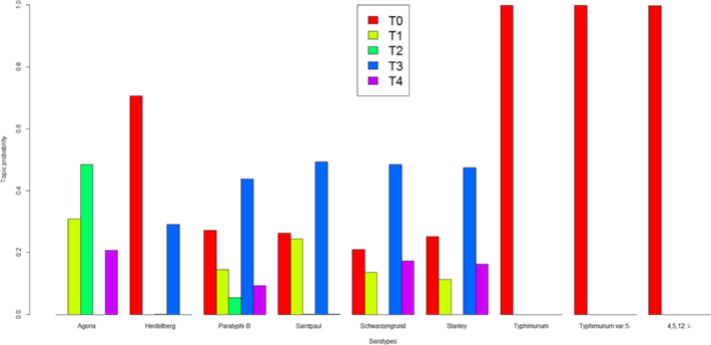
The topic distribution among different serotypes when the topic number is set to five. The five topics and the average probabilities for each serotype were calculated and the colored bars represent topics T0 to T4, respectively

LDA-derived topics classify a group of words which share the similar characteristics. Table 1 lists the top 10 most probable words in each of the five topics when the number of topics was set to 5 (T0, T1, T2, T3, and T4). In each topic, the words were shown in the order of the probabilities, from high to low. Each topic had its unique word composition, and the top 10 words among the five topics were different. Each strain had a unique file consisting of various topics and corresponding probabilities (Additional file 2: Table S2). Strains of the same serotype exhibited similar topic mixture coefficients (Additional file 2: Table S2). We have grouped the strains by serotypes and calculated the average probabilities of the topics for each serotype (Fig. 3). The topic distributions varied with serotypes. Three serotypes, Typhimurium, Typhimurium var. 5-, and 4,[5],12:i:-, harbored only one topic T0 (shown in red bar); while the absences of topics T0 and T3 differentiated the serotype Agona from the other eight serotypes. Serotype Heidelberg was unique, lacking topics T1, T2, and T4. Serotype Saintpaul distinguished itself from serotypes Schwarzengrund and Stanley by missing topic T4. The various topic distributions could be used to differentiate serotypes.

**Table 1:** Topic-10 most probable words obtained when topic number is set to five

Topic ID	Topic-10 most probable words
Topic 0	1724C; 1662G; 1654A; 456C; 434C; 415G; 383G; 382G; 372C; 361A
Topic 1	1443 T; 1020G; 842C; 827C; 706A; 616C; 1594 T; 1532-; 1433C; 1416A
Topic 2	1137G; 1010 T; 1676A; 1520 T; 1378G; 1354A; 1314 T; 1297G; 1294G; 1245A
Topic 3	1115C; 656 T; 913G; 1100G; 1059C; 1027-; 1026-; 746 T; 705 T; 660G
Topic 4	1835 T; 1748 T; 1627A; 1626A; 1549 T; 1511C; 1469 T; 1348A; 1317 T; 1147A

### Two-way hierarchical clustering

**Fig. 4:**
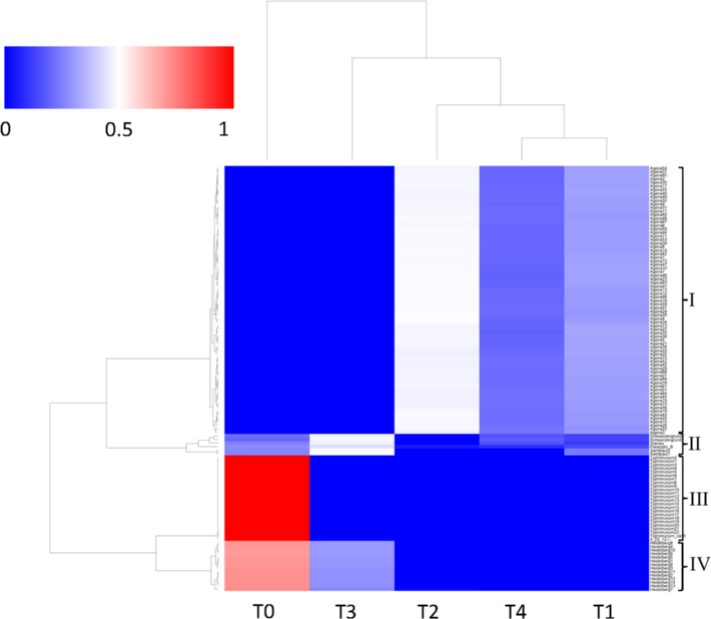
Two-way hierarchical clustering analysis of the LDA-derived strain-topic matrix. The complete-link hierarchical clustering algorithm was applied on the Euclidean distance measures of the topics in any two of the strains in the dataset. The heat map shows that the 119 strains are clustered into four groups (I to IV groups). I: Agona; II: Saintpaul, Paratyphi B, Schwarzengrund and Stanley; III: Typhimurium, Typhimurium var.5- and 4,[5],12:i:- ; IV: Heidelberg. The color histogram from blue to red shows the value of the topic weights of the strains ranged from 0 to 1

The two mixtures derived from LDA (Fig. 1 (e) and (f)) provided vast probabilities for data mining. Hierarchical clustering analysis was first conducted on the strain-topic mixtures (Fig. 1(f)) of all 119 strains in the data set to identify the relationships between the strains, serotypes, and the obtained five topics (Fig. 4). The heat map shows that the strains with the same serotype were grouped together and all 119 strains were clustered into four groups (I to IV): 75 strains of Agona expressing f, g, and s factors of the *fliC* gene were clustered in group I; 24 strains of serotypes Typhimurium, Typhimurium var.5- and 4,[5],12:i:- with *i* factor of the *fliC* gene were clustered together in group III; 14 strains of Heidelberg with factor “r” of the *fliC* gene were grouped into group IV; and the six strains from serotypes Schwarzengrund, Stanley, Paratyphi B, and Saintpaul were in group II. The results are consistent with those shown in Fig. 3. Serotypes Agona and Heidelberg, as well as the group of three serotypes Typhimurium, Typhimurium var.5- and 4,[5],12:i:-, have unique and distinguishable topic compositions; while the serotypes Schwarzengrund, Stanley, Paratyphi B, and Saintpaul share more or less similar topic compositions. The clustering patterns on the data matrix derived from the topic modeling reflected the genetic truth of the serotype determinant *fliC* gene. According to the CDC’s annual report [38], I 4,[5],12:i:- is the monophasic variant of Typhimurium (formula I 4,[5],12:i:1,2) and lacks the second phase H antigen 1,2. In surveillance reports, Typhimurium var. 5- has been considered an O:5-negative variant of Typhimurium or reported as Typhimurium [38]. Strains of all three serotypes express factor *i* of the *fliC* gene and were classified in one group. Figure 4 clearly showed not only the topic distributions among serotypes (as shown in Fig. 3), but also the relationships between the topics. The topic distributions and SNPs compositions in topics were confirmed to be important features that could be used to characterize bacterial strains and distinguish serotypes.

### Distance matrix analysis

**Fig. 5:**
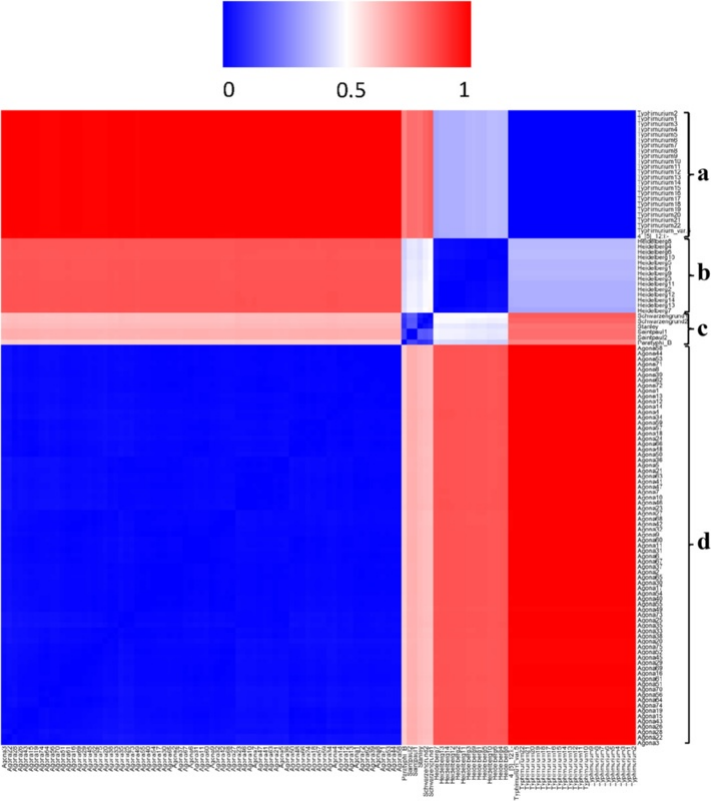
Distance matrix of 119 strains with topic number set to 5. The heat map shows the distance matrix performed on topic mixture representations of the 119 strains. The color histogram from blue to red indicates various degrees of similarity of topic mixtures between every pair of strains. Four groups are visualized. Group A: Typhimurium, Typhimurium var.5- and 4,[5],12:i:-; Group B: Heidelberg; Group C: Saintpaul, Paratyphi B, Schwarzengrund and Stanley; Group D: Agona

Distance matrix analysis was performed on the LDA-derived (topic number set to 5) strain-topic matrix of the 119 strains (Fig. 5). Colors ranging from blue to red indicate various degrees of similarity of topic mixtures between every pair of strains. The blue squares in the diagonal, which are distinguishable from the other squares, represent concordance among the strains within the same serotypes. All of the 119 strains were classified into four subgroups (A, B, C and D). Strains of Typhimurium and its variants were grouped into subgroup A, while strains of Heidelberg were grouped into subgroup B. The distances of the strains between subgroups A and B are much smaller (light blue), compared to the distances to the strains in other subgroups (light red to red). The results show that serotypes Heidelberg, Typhimurium and its variants share the same *fliC* factor i and are genetically close to each other. Strains of serotype Agona were clustered into subgroup D, containing similar topics. The distances of Agona strains to other serotype strains are much further. The results shown in Fig. 3, 4 and 5 are concordant with each other.

**Fig. 6:**
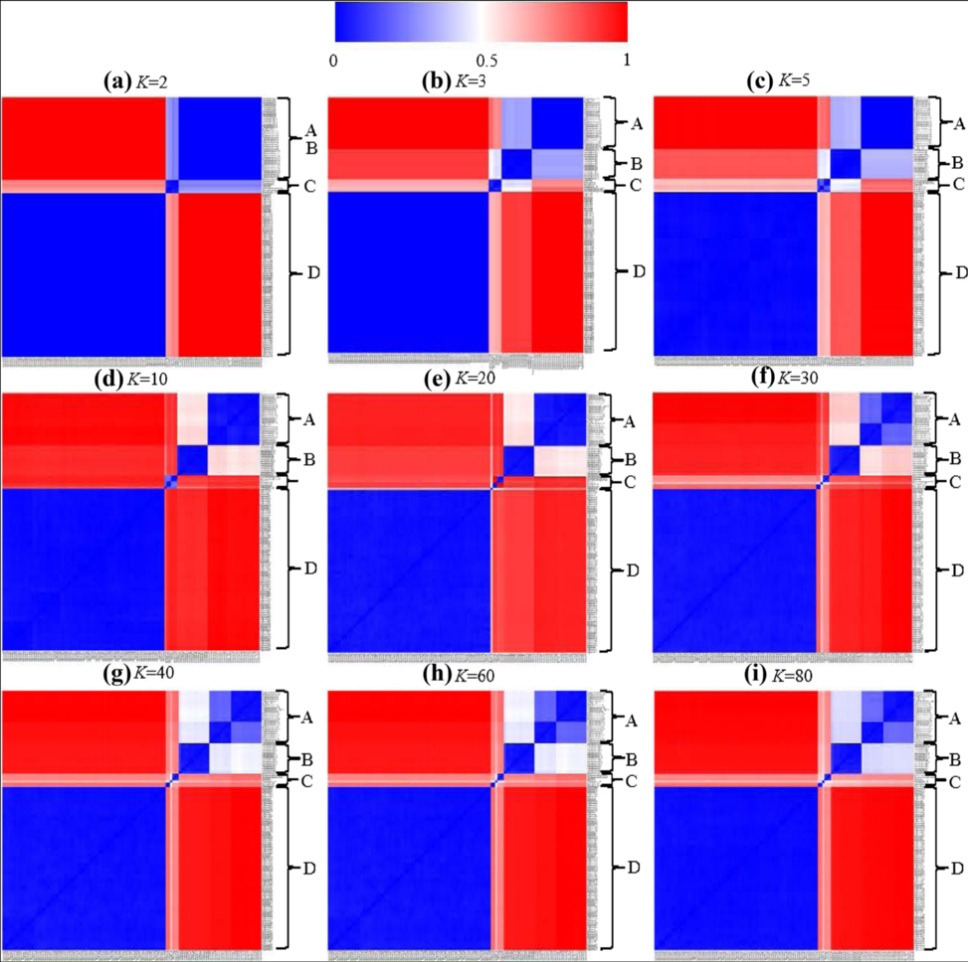
Distance matrix of 119 strains with various topic numbers. Nine heat maps show the distance matrices when the topic number K is set to 2 (a), 3 (b), 5 (c), 10 (d), 20 (e), 30 (f), 40 (g), 60 (h), and 80 (i), respectively. The analysis was performed on the topic mixture representations of the 119 strains by the same method as shown in Fig. 4. The color histogram from blue to red indicates various degrees of similarity of topic mixtures between every pair of strains. Four groups are visualized. Group A: Typhimurium, Typhimurium var.5- and 4,[5],12:i:-; Group B: Heidelberg; Group C: Saintpaul, Paratyphi B, Schwarzengrund and Stanley; Group D: Agona

We also ran the LDA algorithms on the SNPs corpus with the topic numbers (*K*) set to 2, 3, 5, 10, 20, 40, 60, and 80, respectively, to clarify the effect of topic number variation on the resulted biological importance. Data mining was then performed on the obtained strain-topic mixtures representing the SNPs in the *fliC* gene of 119 *Salmonella* strains. Figure 6 shows the heat map of the distance matrices performed at the LDA outputs with various topic numbers. A, B, C, and D represent the same serotype groups as shown in Fig. 5. When *K* is set to two, the groups A and B cannot be separated, and the distance between the groups A/B and C is relatively close (Fig. 6(a)), indicating that the strain-topic matrices are not able to provide sufficient information to distinguish the serotypes included in groups of A, B and C. When *K* is increased, the blue squares in the diagonal increase and become more differentiated from each other (Fig. 6(b-g)) until they are stable (Fig. 6(h-i)). The groups A and B were separated from each other when the topic number was set to three (Fig. 6(b)). When *K* was increased to 20, the strains in subgroup C were classified into three groups (Fig. 6(e)) representing three groups of serotypes. The strains in group A were classified into two small closely related subgroups when *K* was set to 30 (Fig. 6(f)) or more, indicating the existing two patterns of serotype Typhimurium var.5- and 4,[5],12:i:-. The pattern differentiation and subgroup classification remain the same when the topic number is set to 60 or more (Fig. 6(h-i)). Therefore, we consider that the strain-topic matrices derived from the LDA algorithm best distinguished the serotype-determinant *fliC* gene when the topic number was set to 30 for this NGS dataset.

### Model evaluation

We compared the performance and accuracy of our proposed topic modeling method with Hamming method, which is one of the existing methods on sequence similarity analysis, by clustering analysis and classification. Table 2 shows the NMI and ARI values of different methods which were applied to evaluate the clustering results. It is obvious that both NMI and ARI measurements were the highest when *k*-means clustering analysis was applied on LDA-derived sample-topic matrix with topic number set to 30, indicating the better accuracy of topic modeling on similarity detection. However, when topic number was set to 5, the clustering quality was not satisfactory due to the insufficient distinguishment among serotypes. The results are consistent with those in Fig. 6 (c). The distinction between different serotypes becomes more significant when the topic number increases from 2 to 30. Five topics were not enough to distinguish the samples with serotypes Paratyphi B, Saintpaul, Schwarzengrund and Stanley. Therefore, the clustering quality was worse than that when 30 topics were applied. In addition, it was noticed that when Hamming distance was used to calculate the sequence similarities, PCA feature extraction had no obvious effect on the qualities of *k*-means clustering, comparing the similar ARI and NMI values of *k*-means, PCA(2) + *k*-means, and PCA(5) + *k*-means, inferring that topic modeling was more efficient than PCA on this dataset even as a dimension reduction tool. Comparing the results of various methods, the method of “highest probable topic assignment” performed the worst, indicating that the highest probable topic only is not sufficient to describe the distinctions among samples.

**Table 2:** Comparison the results of clustering

Method^a^	ARI	NMI
*k*-means	0.9232	0.8861
PCA(2) + *k*-means	0.9202	0.8547
PCA(5) + *k*-means	0.9322	0.8547
LDA(5)	0.5325	0.4209
LDA(30)	0.8634	0.7946
LDA(5) + *k*-means	0.4301	0.713
LDA(30) + *k*-means	**0.9543**	**0.912**

^a^ *k*-means: traditional *k*-means applying on VSM format of dataset, using Hamming distance

PCA(2) + *k*-means: traditional *k*-means applying on 2 feature matrix obtained by PCA

PCA(5) + *k*-means: traditional *k*-means applying on 5 feature matrix obtained by PCA

LDA(5): “highest probable topic assignment” by LDA with 5 topics

LDA(30): “highest probable topic assignment” by LDA with 30 topics

LDA(5) + *k*-means: traditional *k*-means applying on sample-topic matrix by LDA with 5 topics

LDA(30) + *k*-means: traditional *k*-means applying on sample-topic matrix by LDA with 30 topics

Note that PCA(2) and PCA(5) exhibited better clustering qualities than PCA(10) and PCA(30), and are shown in the table

Bold numbers indicate the best results among various methods

Table 3 shows the classification results using both SVM and RF algorithms to compare the proposed topic modeling method and Hamming method. Overall, the LDA derived sample-topic matrix with 30 topics had higher classification accuracies than the matrix from Hamming method. Especially, RF algorithm reached 100 % accurate prediction on the sample-topic matrix with 30 topics.

**Table 3:** Comparison the results of classification

Data Format	SVM	RF
VSM	0.8462	0.9573
Sample-topic matrix(5)	0.9573	0.9829
Sample-topic matrix(30)	**0.9658**	**1**

Bold numbers indicate the best results among various methods

### Biomarker identification and visualization

**Fig. 7:**
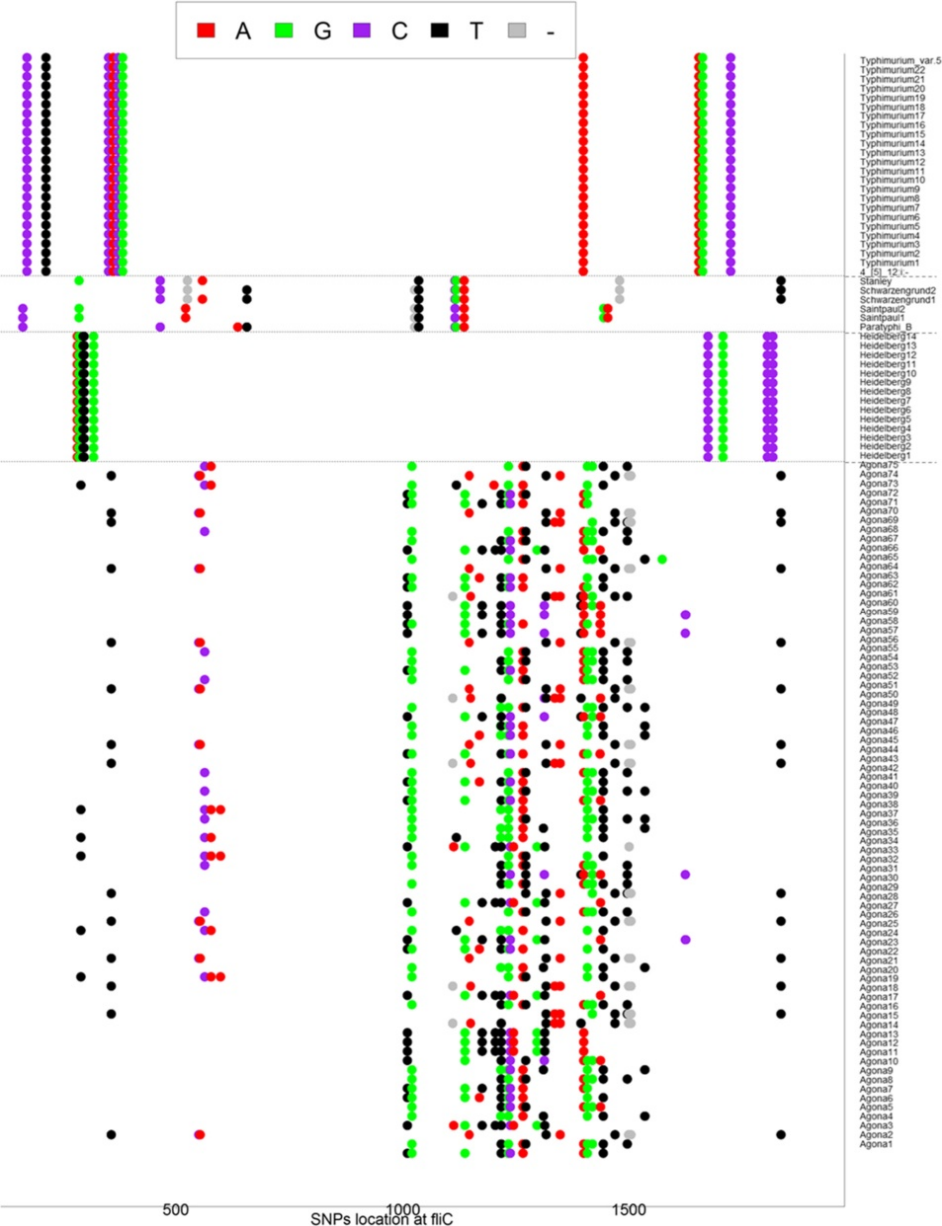
SNPs differentiation and locations in 119 strains. The identified SNPs and their relative locations in the *Salmonella fliC* gene were marked. The red, green, purple, black and grey dots represent A, T, G, C and deletions, respectively

The LDA-derived strain-topic and topic-word matrices could also be combined to analyze the SNPs in the *fliC* gene. The LDA algorithm was run on the text corpus of the *fliC* SNPs from 119 strains with the topic number set to five, resulting in two matrices: strain-topic matrix (119 × 5), and topic-SNP matrix (5 × 2379). We multiplied the two matrices and generated a new matrix of strain-SNPs, from which the top 10 SNPs were then collected for each strain. A sketch was plotted to show the dissemination of these highly emerged SNPs in 119 strains (Fig. 7). The colored dots represent SNPs of A, T, C, G, and deletions as well as their locations in the *fliC* gene. The dissemination of the top ten SNPs for the 119 strains was shown to be serotype-dependent. Especially, the top 10 SNPs are identical for the strains of the same serotypes of Heidelberg, Typhimurium group (including Typhimurium, Typhimurium var.5- and 4,[5],12:i:-), Schwarzengrund and Saintpaul. The strains of serotype Agona exhibit more diversity on SNPs dissemination, with a majority of the SNPs located between 1000–1500 bp. The accuracies of these SNPs in the 119 strains have been confirmed by the sequences from NCBI (Additional file 1: Table S1). The information about the commonalities and diversities in and between serotypes shown in Fig. 7 provides vast possibilities for potential biomarker discrimination, strain evolution, source tracking, and genomic knowledge interpretation (more details in Discussion).

## Discussion

In this study, we proposed a novel procedure to analyze NGS data and discussed its computational implementation as an integrated tool to analyze a target gene’s diversity from the whole genome sequence reads. The key element of the procedure is the application of topic modeling and its integration with current sequence analysis tools and data mining methods. Topic modeling has been widely used in large dataset analysis [10, 14, 15]. Its algorithms analyse the words of documents to discover the themes that pervade a large collection of documents [39]. The rational for incorporating topic modeling on NGS data analysis was based on the fact that the four nucleotides as well as their orders in NGS sequences could be treated as “words”, therefore, the genetic information in sequences was translated and exhibited as “a bag of words”. Two matrices generated by the algorithms of topic modeling provide huge potential applications by combining with various data mining methods for different purposes. The main motivation of this study was to develop the text mining and data mining methods of NGS data analysis for better understanding the genetic diversity of bacterial pathogen populations with high rates of nucleotide substitutions.

The *Salmonella fliC* gene was used as a case study to show one of the applications of the proposed procedure on genetic diversity and biomarker clarification for bacterial pathogen populations. Epidemiological investigations of *Salmonella* infections in humans and animals have depended on serotyping of the cultivated isolates for more than 70 years [40, 41]. Currently there are more than 2,500 serotypes within *S. enterica* and *S. bongori* due to the various combinations of 46 O antigens and 85 H antigens [41]. The limitations of traditional serotyping have stimulated the request for developing new DNA-based serotyping methods. Multiple methods have been investigated for their abilities as a replacement for *Salmonella* serotyping, such as PFGE (Pulsed-Field Gel Electrophoresis) [42–45], ribotyping [46], repetitive extragenic palindromic sequence-based PCR (rep-PCR) [46], microsphere-based liquid arrays, and Multilocus Sequence Typing (MLST) [47]. These methods are feasible for some serotypes, but lack widespread adoption and might misidentify a newly emergent serotype [48]. The nature of unbiased NGS approaches allows numerous applications in comprehensive pathogen detection, infectious disease diagnosis, outbreak investigation and surveillance at a global level [49]. However, the latest applications have mostly relied on phylogenetic clustering and comparisons based on whole genome reads [26, 49, 50]. It is challenging to globally estimate a targeted functional encoding gene’s diversity in bacterial pathogen populations from whole genome reads. Before the cost-effective NGS sequencing technologies became available, this was approached by sequencing PCR-amplified fragments of cultivated isolates, which was indirect, usually biased and inaccurate due to genetic diversity, oligonucleotide primer design, and experimental errors.

The proposed procedure is designed to work on NGS reads or contigs from the NCBI bio-project NGS sequence database (Additional file 1: Table S1) or any other data source, and the specific target gene-related reads or contigs (e.g. *Salmonella fliC*-related gene fragments) were retrieved for the following alignment. The SNPs we identified in this study were different from the original designation, in which all the variations in the same location were included (Fig. 1(c)). Therefore, the “bag of SNPs” (Fig. 1(d)) covered all possible variations in the *fliC*-coding region. These variations, as well as their locations, could be used as characteristics to distinguish strain similarities or identify new mutants (Fig. 7). Moreover, the original data of ATGC sequence reads were transferred into a file of texts on which the text mining/data mining tools can be applied for deep analyses. In the example used in this study, by using the strain-topic matrix derived from topic modeling, we investigated the relationships and similarities between 119 strains and nine serotypes by two-way hierarchical cluster analysis and distance matrix analysis (Fig. 4,5, and 6). Since the *Salmonella fliC* is the coding gene for *Salmonella* phase 1 antigen, and is considered one of the *Salmonella* serotype determinant genes [20], the diversity of *Salmonella fliC* and the relationship with the corresponding serotypes (Fig. 3, 4, 5, 6, and 7) more accurately reflected gene-phenotype relationships than the whole genome sequence phylogenetic trees. The nucleotide-level *Salmonella fliC* gene diversity can be potentially used as the biomarker for serotype screening (Fig. 7). The more NGS sequences are added from more strains and serotypes, the higher the accuracy of the biomarker will be.

In this study, Gibbs sampling was used to maximize the probabilities of the obtained text corpus. An important property of the Gibbs sampling approach is its convergent efficiency. If it takes too many iterations to converge, Gibbs sampling approach will not be a feasible tool for real applications. To test if Gibbs sampling converged fast in the LDA model, we computed the likelihood of the model consisting of both a Dirichlet-multinomial for the SNPs in each topic and a Dirichlet-multinomial for the topics in each strain. The formula used for model likelihood is shown in Eq. 2. The larger the value of the model likelihood is, the better the model that is obtained. Gibbs sampling in LDA converges once the value is stable.

2 $$ LL(model)={\displaystyle {\prod}_{s=1}^s\left\{\frac{\varGamma \left({\displaystyle {\sum}_{i=1}^k ai}\right)}{\varGamma \left({\displaystyle {\sum}_{i=1}^k\left({a}_i+{n}_{si}\right)}\right)}{\displaystyle {\prod}_{i=1}^k\frac{\varGamma \left({a}_i+{n}_{si}\right)}{\varGamma \left({a}_i\right)}}\right\}\cdot {\displaystyle {\prod}_{k=1}^k\left\{\frac{\varGamma \left({\displaystyle {\sum}_{j=1}^v\beta j}\right)}{\varGamma \left({\displaystyle {\sum}_{j=1}^v\left({\beta}_j+{n}_{kj}\right)}\right)}{\displaystyle {\prod}_{j=1}^v\frac{\varGamma \left({\beta}_j+{n}_{kj}\right)}{\varGamma \left({\beta}_j\right)}}\right\}}} $$

**Fig. 8:**
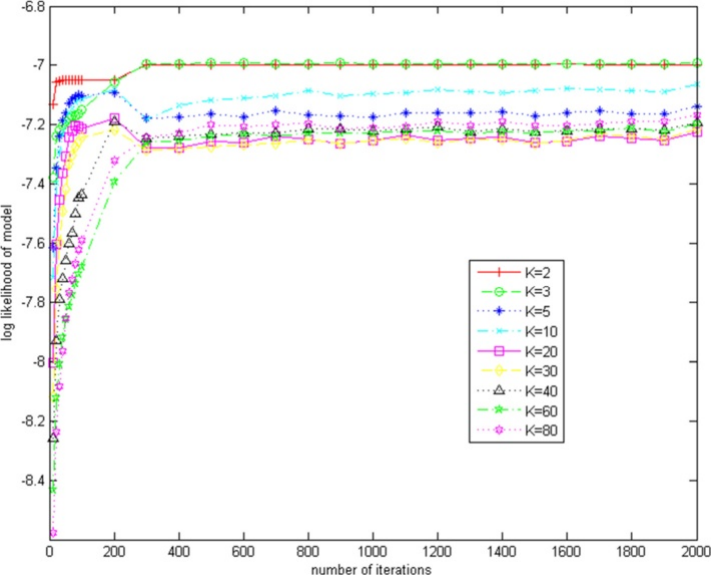
Gibbs sampling convergence test. The likelihood of the model, consisting of both a Dirichlet-multinomial for the SNPs in each topic and a Dirichlet-multinomial for the topics in each strain, was computed when the topic number was set to various numbers. The log of Eq. 2 of every 100 iterations was calculated (every 10 iterations for the first 100)

Here in Eq.2, *V* represents the size of the vocabulary (number of different words in the corpus). The first part in right-hand side of Eq.2 is the Dirichlet-multinomial for the topics in *S* documents and *n*_*si*_ represents the number of topics *i* picked in document *s*; The second part in right-hand side of Eq.2 is the Dirichlet-multinomial for the words in *K* topics and *n*_*kj*_ represents the number of words *j* picked in topic *k*. The log of Eq. 2 for every 100 iterations was calculated (every 10 iterations for the first 100). Fig. 8 shows the results of the convergence test of Gibbs sampling in the LDA algorithm with the number of topics set at different numbers. The log likelihood of the model increases fast in the first 100 iterations, then becomes stable after about 300 iterations (500 iterations when the topic number is three). Gibbs sampling reaches the convergence around 300, indicating that the running of the LDA algorithm is fast to reach the best result. Therefore, the proposed procedure and the implemented tool will be a fast, efficient method and workflow for NGS data analysis and data mining.

Our procedure could be applied on NGS datasets for genetic diversity identification and biomarker development on various functional genes and gene clusters in various biological and biomedical areas. The proposed procedure has implemented the topic modeling algorithms and data mining algorithms in the NGS data analysis, and the resulting sample-topics and topic-words matrices provide huge possibilities for data mining and interpretation. Furthermore, the algorithms in the procedure are designed to accommodate billions of NGS reads if the users’ computer capacity allows. Therefore, the tool we developed in this study is suitable for overcoming difficulties in big NGS data analysis in biological and medical fields. We expect this procedure to be an efficient tool to cope with high complexity and huge volumes of sequence data for elucidating genetic information, gene-phenotype relationships and biomarker identification. The tool has the potential to give a more complete view of the evolutionary dynamics of the bacterial population.

## Conclusions

We have reported a novel procedure to analyze next-generation sequencing data by introducing topic modeling, which is an active research field in machine learning and has been mainly used as an analytical tool to structure large text corpora for data mining. Four major steps are included in this procedure: NGS data retrieval, preprocessing, topic modeling, and data mining using Latent Dirichlet Allocation (LDA) topic outputs. The performance was evaluated by a case study of the NGS data set of the *Salmonella enterica* strains. The results illustrate that the implementation of topic modeling in NGS data analysis provides a new way to elucidate genetic information from NGS data, and identify the gene-phenotype relationships and biomarkers, especially in the era of biological and medical big data.

### Ethics approval and consent to participate

Not applicable.

### Availability of data and materials

The original whole genome sequences of 119 strains of *Salmonella* O antigen group B were retrieved from the NCBI database, including 75 strains of S. Agona, 14 strains of S. Heidelberg, one strain of S. Paratyphi B, two strains of S. Saintpaul, two strains of S. Schwarzengrund, one strain of S. Stanley, 22 strains of S. Typhimurium, one strain of S. Typhimurium var.5-, and one strain of S. 4, 12:i:-. The WGS Accession Numbers, Sequence Bioproject Numbers, and strain names in NCBI database are listed in Additional file 1: Table S1.

## Additional files

Additional file 1: Table S1. Metadata of 119 samples used in this study. (DOCX 25 kb) Additional file 2: Table S2. Topic mixtures of 119 samples with 5 topics. (DOCX 29 kb)

## Abbreviations

ARI: adjusted rand index
BLAST: basic local alignment search tool
LDA: Latent Dirichlet Allocation
LOOCV: leave one out cross validation
MCMC: Markov chain Monte Carlo
MLST: multilocus sequence typing
MSA: multiple sequence alignment
NCBI: national center for biotechnology information
NGS: next generation sequencing
NMI: normalized mutual information
PFGE: pulsed-field gel electrophoresis
RF: random forest
SNPs: single nucleotide polymorphisms
SVM: support vector machine
VSM: vector space model
WGS: whole genome sequence
