# Erratum to: A novel procedure on next generation sequencing data analysis using text mining algorithm

**DOI:** 10.1186/s12859-016-1156-9

**Published:** 2016-08-03

**Authors:** Weizhong Zhao, James J. Chen, Roger Perkins, Yuping Wang, Zhichao Liu, Huixiao Hong, Weida Tong, Wen Zou

**Affiliations:** 1 Division of Bioinformatics and Biostatistics, National Center for Toxicological Research, U.S. Food and Drug Administration, 3900 NCTR Road, HFT-20, Jefferson, AR 72079 USA; 2 College of Information Engineering, Xiangtan University, Xiangtan, Hunan Province China

## Erratum

After publication of the original article [1] it was brought to our attention that the following was incorrectly placed under subheading ‘3. Classification analysis and comparison’ of subsection ‘Evaluation of topic modeling performance’ of the ‘Methods’ section:

*Topic model-derived clustering method [33] was applied, in which LDA was utilized as a feature reduction approach for cluster analysis. The LDAderived topics were considered as the new features of datasets. The sample-topic matrix (Fig. 1(f)) was treated as a new representation of the original dataset. Based on the sample-topic matrix (topic number was chosen as 5 and 30, respectively), conventional clustering algorithms, such as k-means, was used for the clustering analysis. The number of clusters was set as 7 in the k-means method due to 7 different serotypes in the dataset. While in comparison, k-means algorithm was also applied on VSM matrix using Hamming Distance similarities. For further comparison, due to the dimension reduction of topic modeling approach, the traditional tool of PCA was used to reduce features (Numbers of 2, 5, 10 and 30 were randomly selected as the reduced features, respectively) of VSM matrix followed by the k-means cluster analysis. Moreover, clustering by only LDA referred as “highest probable topic assignment” [33] (5 and 30 topics were used) was also used for comparison. In “highest probable topic assignment”, the LDA-derived topics were made as the clusters of the dataset. Then, each sample was assigned to the cluster (Topic) with the highest probability in the row of the sample-topic matrix. To interpret the clustering results obtained by the k-means algorithm, samples in each cluster were labeled as the dominant serotype of the samples in the cluster. The predicted labels of samples were compared with the true labels (serotypes) to evaluate the clustering quality. The clustering results were evaluated by Normalized mutual information (NMI) [34] and Adjusted Rand Index (ARI) [35]. NMI and ARI are two external validation metrics to evaluate the quality of clustering results with respect to the given true labels of datasets. The range of NMI and ARI values is 0–1. In general, the larger the value is, the better the clustering quality is.*

This passage belongs under subheading ‘2. Cluster analysis and result comparison’ of subsection ‘Evaluation of topic modeling performance’ of the ‘Methods’ section.
