# Supplementary material for: A novel procedure on next generation sequencing data analysis using text mining algorithm
Source: BMC Bioinformatics. 2016 May 13;17:213. doi: 10.1186/s12859-016-1075-9 (PMC4866036; doi:10.1186/s12859-016-1075-9)
Supplement: Additional file 1: Table S1. — Metadata of 119 samples used in this study. (DOCX 25 kb) [file 12859_2016_1075_MOESM1_ESM.docx]

Suppl. Table S1. Metadata of 119 samples used in this study.

| **Labels in this study** | ***Salmonella* *enterica* subsp. *enterica* *serovar* and strain** | **WGS Accession number** | **Bioproject** | **Length of *fliC* (bp)** | ***fliC* factor** |
| --- | --- | --- | --- | --- | --- |
| **Agona1** | Agona str. 01.O.05 | CATQ00000000 | PRJEB1064 | 1,518 | f,g,s |
| **Agona2** | Agona str. 02.O.05 | CATP00000000 | PRJEB1065 | 1,518 | f,g,s |
| **Agona3** | Agona str. 03.O.05 | CATO00000000 | PRJEB1066 | 1,518 | f,g,s |
| **Agona4** | Agona str. 04.O.05 | CATN00000000 | PRJEB1067 | 1,518 | f,g,s |
| **Agona5** | Agona str. 05.O.06 | CATM00000000 | PRJEB1068 | 1,518 | f,g,s |
| **Agona6** | Agona str. 06.O.05 | CATL00000000 | PRJEB1069 | 1,518 | f,g,s |
| **Agona7** | Agona str. 07.O.05 | CATK00000000 | PRJEB1070 | 1,518 | f,g,s |
| **Agona8** | Agona str. 08.A.05 | CATJ00000000 | PRJEB1071 | 1,518 | f,g,s |
| **Agona9** | Agona str. 09.F.08 | CATF00000000 | PRJEB1072 | 1,518 | f,g,s |
| **Agona10** | Agona str. 10.A.05 | CATR00000000 | PRJEB1073 | 1,518 | f,g,s |
| **Agona11** | Agona str. 11.A.05 | CATE00000000 | PRJEB1074 | 1,518 | f,g,s |
| **Agona12** | Agona str. 12.A.06 | CATD00000000 | PRJEB1075 | 1,518 | f,g,s |
| **Agona13** | Agona str. 13.E.05 | CATC00000000 | PRJEB1076 | 1,518 | f,g,s |
| **Agona14** | Agona str. 14.E.05 | CATB00000000 | PRJEB1077 | 1,518 | f,g,s |
| **Agona15** | Agona str. 15.H.03 | CATA00000000 | PRJEB1078 | 1,518 | f,g,s |
| **Agona16** | Agona str. 16.H.08 | CATY00000000 | PRJEB1079 | 1,518 | f,g,s |
| **Agona17** | Agona str. 17.H.06 | CASY00000000 | PRJEB1080 | 1,518 | f,g,s |
| **Agona18** | Agona str. 18.H.07 | CASX00000000 | PRJEB1081 | 1,518 | f,g,s |
| **Agona19** | Agona str. 19.F.03 | CASW00000000 | PRJEB1082 | 1,518 | f,g,s |
| **Agona20** | Agona str. 20.H.06 | CASV00000000 | PRJEB1083 | 1,518 | f,g,s |
| **Agona21** | Agona str. 21.H.10 | CASU00000000 | PRJEB1084 | 1,518 | f,g,s |
| **Agona22** | Agona str. 22.H.04 | CAST00000000 | PRJEB1085 | 1,518 | f,g,s |
| **Agona23** | Agona str. 23.F.01 | CASS00000000 | PRJEB1086 | 1,518 | f,g,s |
| **Agona24** | Agona str. 24.H.04 | CASR00000000 | PRJEB1087 | 1,518 | f,g,s |
| **Agona25** | Agona str. 26.F.98 | CATX00000000 | PRJEB1088 | 1,518 | f,g,s |
| **Agona26** | Agona str. 27.O.98 | CASQ00000000 | PRJEB1089 | 1,518 | f,g,s |
| **Agona27** | Agona str. 28.O.08 | CASP00000000 | PRJEB1090 | 1,518 | f,g,s |
| **Agona28** | Agona str. 29.O.08 | CASO00000000 | PRJEB1091 | 1,518 | f,g,s |
| **Agona29** | Agona str. 30.H.04 | CATW00000000 | PRJEB1092 | 1,518 | f,g,s |
| **Agona30** | Agona str. 31.H.09 | CASN00000000 | PRJEB1093 | 1,518 | f,g,s |
| **Agona31** | Agona str. 32.A.00 | CASM00000000 | PRJEB1094 | 1,518 | f,g,s |
| **Agona32** | Agona str. 33.A.05 | CASL00000000 | PRJEB1095 | 1,518 | f,g,s |
| **Agona33** | Agona str. 34.H.09 | CASK00000000 | PRJEB1096 | 1,518 | f,g,s |
| **Agona34** | Agona str. 35.H.08 | CASJ00000000 | PRJEB1097 | 1,518 | f,g,s |
| **Agona35** | Agona str. 36.H.00 | CASI00000000 | PRJEB1098 | 1,518 | f,g,s |
| **Agona36** | Agona str. 37.F.02 | CASH00000000 | PRJEB1099 | 1,518 | f,g,s |
| **Agona37** | Agona str. 38.O.03 | CASG00000000 | PRJEB1100 | 1,518 | f,g,s |
| **Agona38** | Agona str. 39.O.03 | CATV00000000 | PRJEB1101 | 1,518 | f,g,s |
| **Agona39** | Agona str. 40.E.08 | CASE00000000 | PRJEB1103 | 1,518 | f,g,s |
| **Agona40** | Agona str. 41.E.09 | CASF00000000 | PRJEB1102 | 1,518 | f,g,s |
| **Agona41** | Agona str. 42.E.09 | CASD00000000 | PRJEB1104 | 1,518 | f,g,s |
| **Agona42** | Agona str. 43.E.09 | CASC00000000 | PRJEB1105 | 1,518 | f,g,s |
| **Agona43** | Agona str. 44.E.09 | CASB00000000 | PRJEB1106 | 1,518 | f,g,s |
| **Agona44** | Agona str. 45.E.09 | CASA00000000 | PRJEB1107 | 1,518 | f,g,s |
| **Agona45** | Agona str. 46.E.09 | CARZ00000000 | PRJEB1108 | 1,518 | f,g,s |
| **Agona46** | Agona str. 48.E.08 | CARY00000000 | PRJEB1110 | 1,518 | f,g,s |
| **Agona47** | Agona str. 49.E.09 | CATU00000000 | PRJEB1111 | 1,518 | f,g,s |
| **Agona48** | Agona str. 50.E.08 | CARX00000000 | PRJEB1112 | 1,518 | f,g,s |
| **Agona49** | Agona str. 51.E.09 | CARW00000000 | PRJEB1113 | 1,518 | f,g,s |
| **Agona50** | Agona str. 52.F.08 | CARV00000000 | PRJEB1114 | 1,518 | f,g,s |
| **Agona51** | Agona str. 53.F.08 | CARU00000000 | PRJEB1115 | 1,518 | f,g,s |
| **Agona52** | Agona str. 54.O.08 | CART00000000 | PRJEB1116 | 1,518 | f,g,s |
| **Agona53** | Agona str. 55.U.08 | CARS00000000 | PRJEB1117 | 1,518 | f,g,s |
| **Agona54** | Agona str. 56.O.08 | CARR00000000 | PRJEB1118 | 1,518 | f,g,s |
| **Agona55** | Agona str. 57.A.08 | CATT00000000 | PRJEB1119 | 1,518 | f,g,s |
| **Agona56** | Agona str. 58.E.08 | CARQ00000000 | PRJEB1120 | 1,518 | f,g,s |
| **Agona57** | Agona str. 59.F.08 | CARP00000000 | PRJEB1121 | 1,518 | f,g,s |
| **Agona58** | Agona str. 60.O.08 | CARO00000000 | PRJEB1122 | 1,518 | f,g,s |
| **Agona59** | Agona str. 61.O.08 | CARN00000000 | PRJEB1123 | 1,518 | f,g,s |
| **Agona60** | Agona str. 62.H.72 | CARM00000000 | PRJEB1124 | 1,518 | f,g,s |
| **Agona61** | Agona str. 63.H.87 | CARL00000000 | PRJEB1125 | 1,518 | f,g,s |
| **Agona62** | Agona str. 64.H.00 | CARK00000000 | PRJEB1126 | 1,518 | f,g,s |
| **Agona63** | Agona str. 65.H.72 | CARJ00000000 | PRJEB1127 | 1,518 | f,g,s |
| **Agona64** | Agona str. 66.F.99 | CARI00000000 | PRJEB1128 | 1,518 | f,g,s |
| **Agona65** | Agona str. 67.H.09 | CATS00000000 | PRJEB1129 | 1,518 | f,g,s |
| **Agona66** | Agona str. 68.U.05 | CARG00000000 | PRJEB1130 | 1,518 | f,g,s |
| **Agona67** | Agona str. 69.H.06 | CARF00000000 | PRJEB1131 | 1,518 | f,g,s |
| **Agona68** | Agona str. 70.E.05 | CARE00000000 | PRJEB1132 | 1,518 | f,g,s |
| **Agona69** | Agona str. 71.E.05 | CARD00000000 | PRJEB1133 | 1,518 | f,g,s |
| **Agona70** | Agona str. 72.A.52 | CARC00000000 | PRJEB1134 | 1,518 | f,g,s |
| **Agona71** | Agona str. 73.H.09 | CARB00000000 | PRJEB1135 | 1,518 | f,g,s |
| **Agona72** | Agona str. SH08SF124 | ANOT00000000 | PRJNA182122 | 1,518 | f,g,s |
| **Agona73** | Agona str. SH10GFN094 | ANOU00000000 | PRJNA182123 | 1,518 | f,g,s |
| **Agona74** | Agona str. SH11G1113 | ANOS00000000 | PRJNA181255 | 1,518 | f,g,s |
| **Agona75** | Agona str. SL483 | CP001138 | PRJNA20063 | 1,518 | f,g,s |
| **Heidelberg1** | Heidelberg str. 41563 | AJGX00000000 | PRJNA78487 | 1,481 | r |
| **Heidelberg2** | Heidelberg str. 41565 | AJHA00000000 | PRJNA80521 | 1,481 | r |
| **Heidelberg3** | Heidelberg str. 41566 | AJGZ00000000 | PRJNA78489 | 1,482 | r |
| **Heidelberg4** | Heidelberg str. 41573 | AJGY00000000 | PRJNA80513 | 1,481 | r |
| **Heidelberg5** | Heidelberg str. 41578 | CP004086 | PRJNA78477 | 1,482 | r |
| **Heidelberg6** | Heidelberg str. 41579 | AJGW00000000 | PRJNA78479 | 1,482 | r |
| **Heidelberg7** | Heidelberg str. B182 | AJGY00000000 | PRJNA80513 | 1,481 | r |
| **Heidelberg8** | Heidelberg str. CFSAN002069 | CP005390 | PRJNA184142 | 1,482 | r |
| **Heidelberg9** | Heidelberg str. CFSAN00322 | AMBU00000000 | PRJNA162785 | 1,482 | r |
| **Heidelberg10** | Heidelberg str. CFSAN00325 | AMBV00000000 | PRJNA162791 | 1,482 | r |
| **Heidelberg11** | Heidelberg str. CFSAN00326 | AMBW00000000 | PRJNA162793 | 1,482 | r |
| **Heidelberg12** | Heidelberg str. CFSAN00328 | AMBX00000000 | PRJNA162797 | 1,482 | r |
| **Heidelberg13** | Heidelberg str. SL476 | CP001120 | PRJNA20045 | 1,482 | r |
| **Heidelberg14** | Heidelberg str. SL486 | ABEL00000000 | PRJNA20065 | 1,482 | r |
| **Paratyphi_B** | Paratyphi B str. SPB7 | CP000886 | PRJNA27803 | 1,488 | b |
| **Saintpaul1** | Saintpaul str. SARA23 | ABAM00000000 | PRJNA19461 | 1,509 | e,h |
| **Saintpaul2** | Saintpaul str. SARA29 | ABAN00000000 | PRJNA19463 | 1,509 | e,h |
| **Schwarzengrund1** | Schwarzengrund str. CVM19633 | CP001127 | PRJNA19459 | 1,521 | d |
| **Schwarzengrund2** | Schwarzengrund str. SL480 | ABEJ00000000 | PRJNA20071 | 1,521 | d |
| **Stanley** | Stanley str. 06-0538 | ARYV00000000 | PRJNA198795 | 1,521 | d |
| **4_[5]_12:i:-** | Typhimurium str. 08-1736 | CP006602 | PRJNA50689 | 1488 | i |
| **Typhimurium1** | Typhimurium str. 14028S | CP001363 | PRJNA33067 | 1488 | i |
| **Typhimurium2** | Typhimurium str. 798 | CP003386 | PRJNA66023 | 1488 | i |
| **Typhimurium3** | Typhimurium str. D23580 | FN424405 | PRJEA40625 | 1488 | i |
| **Typhimurium4** | Typhimurium str. LT2 | AE006468 | PRJNA241 | 1488 | i |
| **Typhimurium5** | Typhimurium str. SL1344 | FQ312003 | PRJNA50407 | 1488 | i |
| **Typhimurium6** | Typhimurium str. ST1660/06 | AJTU00000000 | PRJNA82323 | 1488 | i |
| **Typhimurium7** | Typhimurium str. ST4/74 | CP002487 | PRJNA56087 | 1488 | i |
| **Typhimurium8** | Typhimurium str. STm1 | AMDX00000000 | PRJNA171774 | 1488 | i |
| **Typhimurium9** | Typhimurium str. STm10 | AMEE00000000 | PRJNA171800 | 1488 | i |
| **Typhimurium10** | Typhimurium str. STm11 | AMEF00000000 | PRJNA171801 | 1488 | i |
| **Typhimurium11** | Typhimurium str. STm12 | AMEG00000000 | PRJNA171802 | 1488 | i |
| **Typhimurium12** | Typhimurium str. STm2 | AMDY00000000 | PRJNA171794 | 1488 | i |
| **Typhimurium13** | Typhimurium str. STm3 | AMEB00000000 | PRJNA171795 | 1488 | i |
| **Typhimurium14** | Typhimurium str. STm4 | AMEC00000000 | PRJNA171796 | 1488 | i |
| **Typhimurium15** | Typhimurium str. STm5 | AMEH00000000 | PRJNA171803 | 1488 | i |
| **Typhimurium16** | Typhimurium str. STm6 | AMED00000000 | PRJNA171797 | 1488 | i |
| **Typhimurium17** | Typhimurium str. STm8 | AMDZ00000000 | PRJNA171798 | 1488 | i |
| **Typhimurium18** | Typhimurium str. STm9 | AMEA00000000 | PRJNA171799 | 1488 | i |
| **Typhimurium19** | Typhimurium str. T000240 | AP011957 | PRJNA45951 | 1488 | i |
| **Typhimurium20** | Typhimurium str. TN061786 | AERV00000000 | PRJNA61789 | 1488 | i |
| **Typhimurium21** | Typhimurium str. U288 | CP003836 | PRJNA157919 | 1488 | i |
| **Typhimurium22** | Typhimurium str. UK-1 | CP002614 | PRJNA63211 | 1488 | i |
| **Typhimurium_var.5** | Typhimurium var.5- str. CFSAN001921 | CP006048 | PRJNA184140 | 1488 | i |
