# Supplementary material for: A novel procedure on next generation sequencing data analysis using text mining algorithm
Source: BMC Bioinformatics. 2016 May 13;17:213. doi: 10.1186/s12859-016-1075-9 (PMC4866036; doi:10.1186/s12859-016-1075-9)
Supplement: Additional file 2: Table S2. — Topic mixtures of 119 samples with 5 topics. (DOCX 29 kb) [file 12859_2016_1075_MOESM2_ESM.docx]

Suppl. Table S2. Topic mixtures of 119 samples with 5 topics.

| **StrainName** | **T0** | **T1** | **T2** | **T3** | **T4** |
| --- | --- | --- | --- | --- | --- |
| **Agona1** | 0.000 | 0.312 | 0.488 | 0.000 | 0.200 |
| **Agona2** | 0.000 | 0.313 | 0.483 | 0.000 | 0.204 |
| **Agona3** | 0.000 | 0.296 | 0.474 | 0.000 | 0.230 |
| **Agona4** | 0.000 | 0.301 | 0.497 | 0.000 | 0.201 |
| **Agona5** | 0.000 | 0.318 | 0.486 | 0.000 | 0.196 |
| **Agona6** | 0.000 | 0.308 | 0.484 | 0.000 | 0.207 |
| **Agona7** | 0.000 | 0.314 | 0.490 | 0.000 | 0.195 |
| **Agona8** | 0.000 | 0.307 | 0.488 | 0.000 | 0.205 |
| **Agona9** | 0.000 | 0.312 | 0.481 | 0.000 | 0.207 |
| **Agona10** | 0.000 | 0.315 | 0.489 | 0.000 | 0.195 |
| **Agona11** | 0.000 | 0.306 | 0.484 | 0.000 | 0.210 |
| **Agona12** | 0.000 | 0.305 | 0.490 | 0.000 | 0.205 |
| **Agona13** | 0.000 | 0.304 | 0.492 | 0.000 | 0.205 |
| **Agona14** | 0.000 | 0.307 | 0.492 | 0.000 | 0.201 |
| **Agona15** | 0.000 | 0.299 | 0.487 | 0.000 | 0.214 |
| **Agona16** | 0.000 | 0.308 | 0.477 | 0.000 | 0.214 |
| **Agona17** | 0.000 | 0.312 | 0.483 | 0.000 | 0.205 |
| **Agona18** | 0.000 | 0.301 | 0.493 | 0.000 | 0.206 |
| **Agona19** | 0.000 | 0.299 | 0.484 | 0.000 | 0.217 |
| **Agona20** | 0.000 | 0.324 | 0.468 | 0.000 | 0.208 |
| **Agona21** | 0.000 | 0.318 | 0.483 | 0.000 | 0.199 |
| **Agona22** | 0.000 | 0.286 | 0.486 | 0.000 | 0.229 |
| **Agona23** | 0.000 | 0.314 | 0.493 | 0.000 | 0.193 |
| **Agona24** | 0.000 | 0.298 | 0.493 | 0.000 | 0.210 |
| **Agona25** | 0.000 | 0.323 | 0.481 | 0.000 | 0.196 |
| **Agona26** | 0.000 | 0.295 | 0.483 | 0.000 | 0.221 |
| **Agona27** | 0.000 | 0.314 | 0.476 | 0.000 | 0.210 |
| **Agona28** | 0.000 | 0.294 | 0.496 | 0.000 | 0.210 |
| **Agona29** | 0.000 | 0.311 | 0.475 | 0.000 | 0.214 |
| **Agona30** | 0.000 | 0.312 | 0.483 | 0.000 | 0.205 |
| **Agona31** | 0.000 | 0.306 | 0.483 | 0.000 | 0.211 |
| **Agona32** | 0.000 | 0.310 | 0.480 | 0.000 | 0.211 |
| **Agona33** | 0.000 | 0.320 | 0.474 | 0.000 | 0.206 |
| **Agona34** | 0.000 | 0.306 | 0.496 | 0.000 | 0.198 |
| **Agona35** | 0.000 | 0.325 | 0.484 | 0.000 | 0.191 |
| **Agona36** | 0.000 | 0.319 | 0.484 | 0.000 | 0.196 |
| **Agona37** | 0.000 | 0.314 | 0.482 | 0.000 | 0.204 |
| **Agona38** | 0.000 | 0.320 | 0.477 | 0.000 | 0.202 |
| **Agona39** | 0.000 | 0.308 | 0.487 | 0.000 | 0.205 |
| **Agona40** | 0.000 | 0.315 | 0.478 | 0.000 | 0.206 |
| **Agona41** | 0.000 | 0.312 | 0.490 | 0.000 | 0.198 |
| **Agona42** | 0.000 | 0.312 | 0.476 | 0.000 | 0.212 |
| **Agona43** | 0.000 | 0.296 | 0.487 | 0.000 | 0.217 |
| **Agona44** | 0.000 | 0.308 | 0.489 | 0.000 | 0.202 |
| **Agona45** | 0.000 | 0.311 | 0.472 | 0.000 | 0.217 |
| **Agona46** | 0.000 | 0.315 | 0.490 | 0.000 | 0.194 |
| **Agona47** | 0.000 | 0.314 | 0.487 | 0.000 | 0.199 |
| **Agona48** | 0.000 | 0.304 | 0.487 | 0.000 | 0.210 |
| **Agona49** | 0.002 | 0.315 | 0.477 | 0.000 | 0.205 |
| **Agona50** | 0.000 | 0.301 | 0.486 | 0.000 | 0.213 |
| **Agona51** | 0.000 | 0.306 | 0.477 | 0.000 | 0.217 |
| **Agona52** | 0.000 | 0.314 | 0.474 | 0.000 | 0.212 |
| **Agona53** | 0.000 | 0.310 | 0.488 | 0.000 | 0.202 |
| **Agona54** | 0.000 | 0.313 | 0.482 | 0.000 | 0.205 |
| **Agona55** | 0.000 | 0.314 | 0.480 | 0.000 | 0.206 |
| **Agona56** | 0.000 | 0.302 | 0.476 | 0.000 | 0.221 |
| **Agona57** | 0.000 | 0.301 | 0.489 | 0.000 | 0.210 |
| **Agona58** | 0.000 | 0.308 | 0.489 | 0.000 | 0.202 |
| **Agona59** | 0.000 | 0.300 | 0.490 | 0.000 | 0.210 |
| **Agona60** | 0.000 | 0.305 | 0.486 | 0.000 | 0.210 |
| **Agona61** | 0.000 | 0.307 | 0.480 | 0.000 | 0.213 |
| **Agona62** | 0.000 | 0.311 | 0.488 | 0.000 | 0.201 |
| **Agona63** | 0.000 | 0.314 | 0.489 | 0.000 | 0.196 |
| **Agona64** | 0.000 | 0.311 | 0.470 | 0.000 | 0.219 |
| **Agona65** | 0.000 | 0.314 | 0.483 | 0.000 | 0.202 |
| **Agona66** | 0.000 | 0.304 | 0.489 | 0.000 | 0.207 |
| **Agona67** | 0.000 | 0.308 | 0.483 | 0.000 | 0.208 |
| **Agona68** | 0.000 | 0.313 | 0.476 | 0.000 | 0.211 |
| **Agona69** | 0.000 | 0.308 | 0.477 | 0.000 | 0.214 |
| **Agona70** | 0.000 | 0.304 | 0.474 | 0.000 | 0.223 |
| **Agona71** | 0.000 | 0.307 | 0.489 | 0.000 | 0.204 |
| **Agona72** | 0.000 | 0.312 | 0.487 | 0.000 | 0.201 |
| **Agona73** | 0.000 | 0.324 | 0.480 | 0.000 | 0.196 |
| **Agona74** | 0.000 | 0.307 | 0.472 | 0.000 | 0.220 |
| **Agona75** | 0.000 | 0.315 | 0.471 | 0.000 | 0.213 |
| **Heidelberg1** | 0.704 | 0.000 | 0.000 | 0.295 | 0.000 |
| **Heidelberg2** | 0.714 | 0.000 | 0.000 | 0.286 | 0.000 |
| **Heidelberg3** | 0.706 | 0.000 | 0.000 | 0.293 | 0.001 |
| **Heidelberg4** | 0.698 | 0.000 | 0.000 | 0.301 | 0.000 |
| **Heidelberg5** | 0.704 | 0.000 | 0.000 | 0.295 | 0.000 |
| **Heidelberg6** | 0.695 | 0.000 | 0.000 | 0.305 | 0.000 |
| **Heidelberg7** | 0.723 | 0.000 | 0.010 | 0.267 | 0.000 |
| **Heidelberg8** | 0.698 | 0.000 | 0.000 | 0.301 | 0.000 |
| **Heidelberg9** | 0.703 | 0.000 | 0.000 | 0.296 | 0.000 |
| **Heidelberg10** | 0.695 | 0.000 | 0.001 | 0.303 | 0.000 |
| **Heidelberg11** | 0.714 | 0.000 | 0.000 | 0.286 | 0.000 |
| **Heidelberg12** | 0.715 | 0.000 | 0.000 | 0.284 | 0.000 |
| **Heidelberg13** | 0.716 | 0.000 | 0.000 | 0.283 | 0.000 |
| **Heidelberg14** | 0.716 | 0.000 | 0.000 | 0.283 | 0.000 |
| **Paratyphi_B** | 0.271 | 0.145 | 0.054 | 0.438 | 0.092 |
| **Saintpaul1** | 0.262 | 0.243 | 0.000 | 0.494 | 0.001 |
| **Saintpaul2** | 0.264 | 0.243 | 0.001 | 0.491 | 0.000 |
| **Schwarzengrund1** | 0.199 | 0.135 | 0.000 | 0.485 | 0.181 |
| **Schwarzengrund2** | 0.220 | 0.135 | 0.000 | 0.482 | 0.163 |
| **Stanley** | 0.251 | 0.113 | 0.000 | 0.474 | 0.162 |
| **4_[5]_12:i:-** | 0.998 | 0.000 | 0.000 | 0.000 | 0.001 |
| **Typhimurium1** | 0.999 | 0.000 | 0.000 | 0.000 | 0.000 |
| **Typhimurium2** | 0.999 | 0.000 | 0.000 | 0.000 | 0.000 |
| **Typhimurium3** | 0.999 | 0.000 | 0.000 | 0.000 | 0.000 |
| **Typhimurium4** | 0.999 | 0.000 | 0.000 | 0.000 | 0.000 |
| **Typhimurium5** | 0.999 | 0.000 | 0.000 | 0.000 | 0.000 |
| **Typhimurium6** | 0.999 | 0.000 | 0.000 | 0.000 | 0.000 |
| **Typhimurium7** | 0.999 | 0.000 | 0.000 | 0.000 | 0.000 |
| **Typhimurium8** | 0.999 | 0.000 | 0.000 | 0.000 | 0.000 |
| **Typhimurium9** | 0.999 | 0.000 | 0.000 | 0.000 | 0.000 |
| **Typhimurium10** | 0.999 | 0.000 | 0.000 | 0.000 | 0.000 |
| **Typhimurium11** | 0.999 | 0.000 | 0.000 | 0.000 | 0.000 |
| **Typhimurium12** | 0.999 | 0.000 | 0.000 | 0.000 | 0.000 |
| **Typhimurium13** | 0.999 | 0.000 | 0.000 | 0.000 | 0.000 |
| **Typhimurium14** | 0.999 | 0.000 | 0.000 | 0.000 | 0.000 |
| **Typhimurium15** | 0.999 | 0.000 | 0.000 | 0.000 | 0.000 |
| **Typhimurium16** | 0.999 | 0.000 | 0.000 | 0.000 | 0.000 |
| **Typhimurium17** | 0.999 | 0.000 | 0.000 | 0.000 | 0.000 |
| **Typhimurium18** | 0.999 | 0.000 | 0.000 | 0.000 | 0.000 |
| **Typhimurium19** | 0.999 | 0.000 | 0.000 | 0.000 | 0.000 |
| **Typhimurium20** | 0.999 | 0.000 | 0.000 | 0.000 | 0.000 |
| **Typhimurium21** | 0.999 | 0.000 | 0.000 | 0.000 | 0.000 |
| **Typhimurium22** | 0.999 | 0.000 | 0.000 | 0.000 | 0.000 |
| **Typhimurium_var.5** | 0.999 | 0.000 | 0.000 | 0.000 | 0.000 |
